# A refined dose metric for nanotoxicology based on surface site reactivity for oxidative potential of engineered nanomaterials†

**DOI:** 10.1039/d5na00104h

**Published:** 2025-02-26

**Authors:** Victor Alcolea-Rodriguez, Felice C. Simeone, Verónica I. Dumit, Lara Faccani, Victoria Toledo, Andrea Haase, Nicolas Coca-López, Raquel Portela, Miguel A. Bañares

**Affiliations:** a CSIC-ICP, Instituto de Catálisis y Petroleoquímica, Spectroscopy and Industrial Catalysis (SpeiCat) Marie Curie 2 28034-Madrid Spain valcolear@gmail.com nicolas.coca@csic.es raquel.portela@csic.es miguel.banares@csic.es; b Department of Chemical and Product Safety, German Federal Institute for Risk Assessment Berlin 10589 Germany veronica.dumit@bfr.bund.de andrea.haase@bfr.bund.de; c National Research Council of Italy (CNR), Istituto per la Scienza, Sostenibilità e Tecnologia dei Materiali Ceramici (ISSMC) Via Granarolo, 64 48018 Faenza RA Italy felice.simeone@issmc.cnr.it lara.faccani@issmc.cnr.it; d Freie Universität Berlin, Institute of Pharmacy Berlin Germany; e Centro de Investigación y Desarrollo en Ciencias Aplicadas “Dr Jorge J. Ronco” CINDECA, CCT La Plata-CONICET, UNLP, CICpBA Calle 47 No. 257 B1900AJK La Plata Buenos Aires Argentina

## Abstract

The increasing production of engineered nanomaterials (ENMs) raises significant concerns about human and environmental exposure, making it essential to understand the mechanisms of their interaction with biological systems to manage the associated risks. To address this, we propose categorizing ENM reactivity using *in chemico* methodologies. Surface analysis through methanol chemisorption and temperature-programmed surface reaction allows for the determination of reactive surface sites, providing accurate estimates of effective ENM doses in toxicity studies. Additionally, antioxidant consumption assays (dithiothreitol, cysteine, and glutathione) and reactive oxygen species (ROS) generation assays (RNO and DCFH_2_-DA) are employed to rank the oxidative potential of ENM surface sites in a cell-free environment. Our study confirms the classification of ZnO NM-110, ZnO NM-111, CuO, and carbon black as highly oxidant ENMs, while TiO_2_ NM-101 and NM-105 exhibit low oxidative potential due to their acidic surface sites. In contrast, CeO_2_ NM-211 and NM-212 demonstrate redox surface sites. SiO_2_ nanomaterials (NM-200 and NM-201) are shown to be inert, with low oxidation rates and minimal reactive surface density, despite their high surface area. Quantifying reactive surface sites offers a refined dose metric for assessing ENM toxicity, advancing safe-by-design nanomaterial development.

## Abstract 2

*In chemico* ENM reactivity assays quantify oxidative rates and reactive surface sites, refining dose metrics for nanotoxicity assessment and enabling safe-by-design nanomaterials.

## Graphical abstract

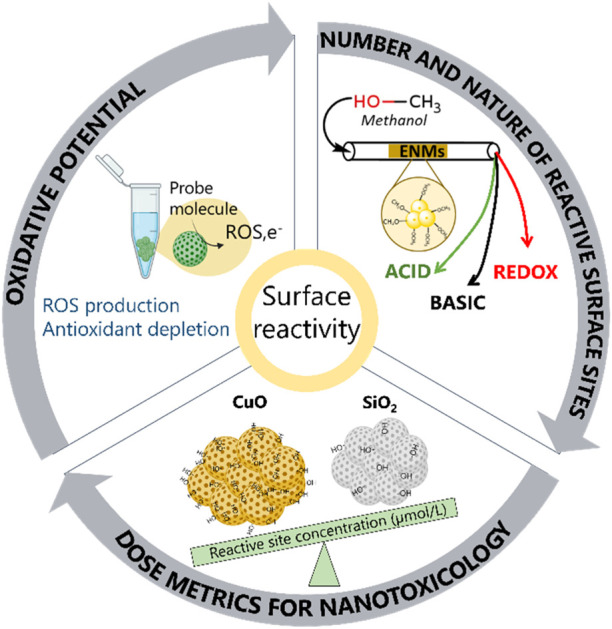

## 1 Introduction

Engineered nanomaterials (ENMs) are increasingly utilized in various sectors, including catalysis, agriculture, cosmetics, electronics, medicine, or food.^1,2^ The massive production of these materials calls for an accurate evaluation of their impact on both human health and the environment.^3^ Understanding their modes of action enables the adoption of Safe and Sustainable by Design (SSbD) approaches to develop nano-enabled products characterized by low risks throughout their life cycle: these approaches make it possible to design ENMs or nano-enabled products with safety considerations integrated early in the product development stage. However, the physico–chemical properties that make them useful also complicate defining their dosage. Unlike bulk chemicals, mass and mole exposure do not always correlate with the ENM exposure dose; different particle sizes dramatically change the fraction of ENM that is exposed. In addition, even for a given ENM, the surface area may have very different populations of reactive sites depending on exposed planes, defects and other variables.^4–7^ For this reason, traditional approaches to chemical safety assessment, which rely on established toxicological protocols, may not fully address the unique challenges presented by ENMs. Furthermore, nano-derived effects as well as the complexity and the speed of new innovations for ENMs require methodological approaches that can be run with less time-consuming procedures.^8^ In addition, the field of toxicology is currently undergoing a significant transformation, moving towards animal-free methods. This context underscores the growing importance of New Approach Methodologies (NAMs) in the evaluation of substances, including ENMs.^9,10^ NAMs, particularly those designed for ENM screening, for instance by measuring ENM reactivity, are valuable tools in the Safe by Design (SbD) framework.

This paper provides a phenomenological description of the reactivity of ENMs by linking physicochemical characteristics to their oxidative potential, a parameter that has been proposed to rank the risk of ENMs.^11–16^ often quantified by FRAS,^17,18^ CPH, and DMPO probes.^19^ These methods, however, are blind to the characteristics of the surface that determine the observed oxidative damage. From a surface-chemistry point of view, it becomes important, in fact, to determine the fraction of the whole surface that is really active, and the nature of this reactive surface. To this goal, we propose a novel, *in chemico* methodology to identify the nature, number, and reactivity of surface sites on an ENM using probe molecules. Gas-phase methanol chemisorption and TPSR are used to quantify the reactive site surface density and their reactive profile. The intrinsic oxidative potential is measured *via* oxidation rates for (1) ROS generation and (2) antioxidant consumption. We employed antioxidant consumption assays (dithiothreitol, cysteine, and glutathione) and reactive oxygen species (ROS) generation assays (detected by *N*,*N*-dimethyl-4-nitrosoaniline, RNO, and 2,7-dichlorodihydrofluorescein diacetate DCFH_2_-DA).^18^ Reactive surface site quantification is used to normalize oxidative potential data, applying the concept of Oxidative Turnover Frequency (OxTOF). This normalization enables a refined and relevant definition of effective dose in terms of reactive surface sites. Therefore, the paper emphasizes that integrating surface reactivity and material chemistry concepts into toxicology protocols may allow an accurate screening and comprehensive assessment of ENM risks.^7^ The study combines the analysis of the reactive surface sites with the data of reactivity to rank a set of 14 common nanomaterials based on the estimations of their oxidative potential normalized to the number of active sites.

## 2 Experimental

### 2.1 Nanomaterials

Fourteen common powdered ENMs were analyzed without any pretreatment: TiO_2_ NM-101 (JRCNM01001a), TiO_2_ NM-105 (JRCNM01005a), CeO_2_ NM-211 (JRCNM02101a), CeO_2_ NM-212 (JRCNM02102a), ZnO NM-110 (JRCNM62101a), ZnO NM-111 (JRCNM01101a), SiO_2_ NM-200 (JRCNM02000a), SiO_2_ NM-201 (JRCNM02001a), MWCNT NM-400 and MWCNT NM-401 were supplied by Joint Research Centre (JRC), while CuO (ref. number: 544868), Mn_2_O_3_ (ref. number: 933791), carbon black (PRINTEX® 90, CB) and Fe_2_O_3_ (ref. number: 544884) were obtained from Sigma-Aldrich (SA). TiO_2_ NM-105 is the same as Aeroxide P25 (Degussa/Evonik), which comprises 73–85% anatase, 14–17% rutile, and 0–13% amorphous titania.^20–22^ The main physicochemical properties of the evaluated NMs are summarized in Table S1.†

### 2.2 Quantification and characterization of reactive surface sites

Reactive sites on the surface of metal oxide nanomaterials were probed by chemisorption of methanol followed by its Temperature Programmed Surface Reaction (TPSR)^23,24^ to identify the number and characteristics of reactive surface sites.^25^ The protocol has been adapted from our previous studies.^26,27^ Briefly, 100–250 mg of NM powder (nanoparticle aggregate size from 25 to 100 μm) were mixed with 0.5 g SiC (black 180, Navarro SiC S.A.) and introduced into a fixed-bed reactor (0.4 cm diameter) to follow the procedure described elsewhere.^26^ Methanol chemisorption at 100 °C (50 °C in the case of CuO and ZnO NM-110 due to their high reactivity) results in the release of H_2_O molecules due to the formation of methoxy species (–OCH_3_) bonding to the reactive surface sites. During linear heating, different reaction products are released according to the reactivity of the surface sites: some unreacted methanol can be released in low-activity sites, acidic sites yield dimethyl ether (CH_3_OCH_3_), redox sites generate formaldehyde (HCHO), and carbon dioxide (CO_2_) can be produced on basic sites (typically above 300 °C) or highly reactive redox sites (below 300 °C). This catalytic clue about the primary reactivity of a nanomaterial is independent of the stability of a dispersion, pretreatments, bio-transformations or ion release. All the evaluated nanomaterials were previously screened for thermal stability in an STA 6000 simultaneous thermal analyzer (PerkinElmer). The thermal stability of the nanotubes up to 450 °C was confirmed by the supplier.^28^

### 2.3 Consumption of antioxidants

To estimate the ability of ENMs to consume antioxidants, we measured the rates at which terminal thiol groups are oxidized in three biologically relevant molecules: (i) dithiothreitol (DTT), (ii) cysteine (Cys) and (iii) glutathione (GSH). DTT is a synthetic di-sulfide used as an unspecific reducing agent. Glutathione is a natural antioxidant characterized by the lowest redox potential that can be found in a cell.^13^ This low redox potential (*i.e.*, high thermodynamic tendency to reduce oxidants) also enables, in principle, the direct oxidation of the S-terminus by species other than ROS (*e.g.*, metal ions).^13,29^ Cysteine is an amino acid, and the oxidation of its terminal –S is relevant for the oxidative damage of proteins.^30^ The details of these acellular procedures are provided in the ESI.†

### 2.4 Generation of reactive oxygen species

We estimated the rates of ROS generation by two acellular approaches. We used *N*,*N*-dimethyl-4-nitrosoaniline (RNO) for the spectroscopic determination of the production of hydroxyl radicals (*i.e.*, OH˙),^31^ and 2′,7′-dichlorodihydrofluorescein diacetate (DCFH_2_-DA) to detect ROS other than *OH.^18^ The specifics of the procedure are detailed in the ESI.† The methodology for DCFH_2_-DA assay is based on the SOP developed by the GRACIOUS EU project (GA760840).

### 2.5 Ranking of ENMs reactivity and investigation of reactivity–toxicity correlations

We used *K*-means and hierarchical dendrogram unsupervised learning algorithms to detect similarity-based descriptors and patterns of reactivity.^32–34^ The main goal of this approach was to group the ENMs based on distance-based metrics or similarity according to their oxidative potential. Reactivity data based on ENM mass, surface and reactive surface sites exposed to the probe molecule were compared in a pair plot. The oxidative potential per reactive surface site obtained for the different probe molecules was compared utilizing Pearson's correlation coefficient.

We used Spearman's coefficient to compare the acellular oxidative potential of ENMs with outcomes of *in vitro* studies by LDH and WST-1 assays in lung cell lines A549 and dTHP-1.^35^ As a toxicological metric, we employed the highest concentration that showed no adverse effects after 24 h exposure; gravimetric concentrations of ENMs (μg mL^−1^) were converted to reactive site concentrations (μmol L^−1^) based on the data obtained with a methanol probe (one methanol molecule titrates one reactive surface site).

## 3 Results

### 3.1 Number and nature of reactive surface sites of ENMs

For each ENM, we evaluated (i) specific surface area (m^2^ g^−1^), (ii) specific number of reactive surface sites (mmol g^−1^, one methanol molecule titrates one reactive surface site), and (iii) reactive site surface density (sites per nm^2^) (Table 1). The ENMs with the largest specific surface area were CB (317 m^2^ g^−1^), MWCNT NM-400 (240 m^2^ g^−1^) and TiO_2_ NM-101 (225 m^2^ g^−1^), whereas the two ZnO, Mn_2_O_3_ and CuO exhibited the lowest values: 9 m^2^ g^−1^ (NM-110), 15 (NM-111), 17 m^2^ g^−1^ and 12 m^2^ g^−1^, respectively. Regarding the number of reactive surface sites, TiO_2_ NM-101 had the highest specific number (2.8 mmol g^−1^), followed by MWCNT NM-400, TiO_2_ NM-105 and MWCNT NM-401, with values between 1.7 and 1.1 mmol g^−1^, whereas zinc oxides, ceria, silica, CuO and Mn_2_O_3_ exhibited a lower specific number of sites (0.1–0.4 mmol g^−1^). These were specific values (per unit mass); however, the analysis of the reactive site surface density, with a variety of values, revealed that the physical specific surface area did not directly correlate with the number of reactive surface sites, as determined by methanol chemisorption. The ENM with the highest reactive site surface density was CuO (22 sites per nm^2^), followed by ZnO NM-110, Fe_2_O_3_ and TiO_2_ NM-105, while the lowest values were those of silica and MWCNT, all below 5 sites per nm^2^.

**Table 1:** Surface descriptors for the tested ENMs. Specific surface area was obtained by using N_2_ adsorption isotherms and the specific number of reactive surface sites by methanol chemisorption. Their ratio is the reactive site surface density. The number of reactive surface sites on carbon black could not be measured due to agglomeration issues in the reactor

Nanomaterial	Specific surface area (m^2^ g^−1^)	Specific number of reactive sites (mmol g^−1^)	Reactive site surface density (reactive site per nm^2^)
TiO_2_ NM-101	225	2.8	7
TiO_2_ NM-105	58	1.1	13
ZnO NM-110	9	0.2	17
ZnO NM-111	15	0.1	5
SiO_2_ NM-200	182	0.3	1
SiO_2_ NM-201	140	0.5	2
CeO_2_ NM-211	76	0.4	6
CeO_2_ NM-212	25	0.4	10
MWCNT NM-400	240^a^	1.7	4
MWCNT NM-401	140^a^	1.1	4
CuO	12	0.4	22
Carbon black	317	NA	NA
Fe_2_O_3_	41	1.0	15
Mn_2_O_3_	17	0.3	11

a Data obtained from the supplier.

**Fig. 1:**
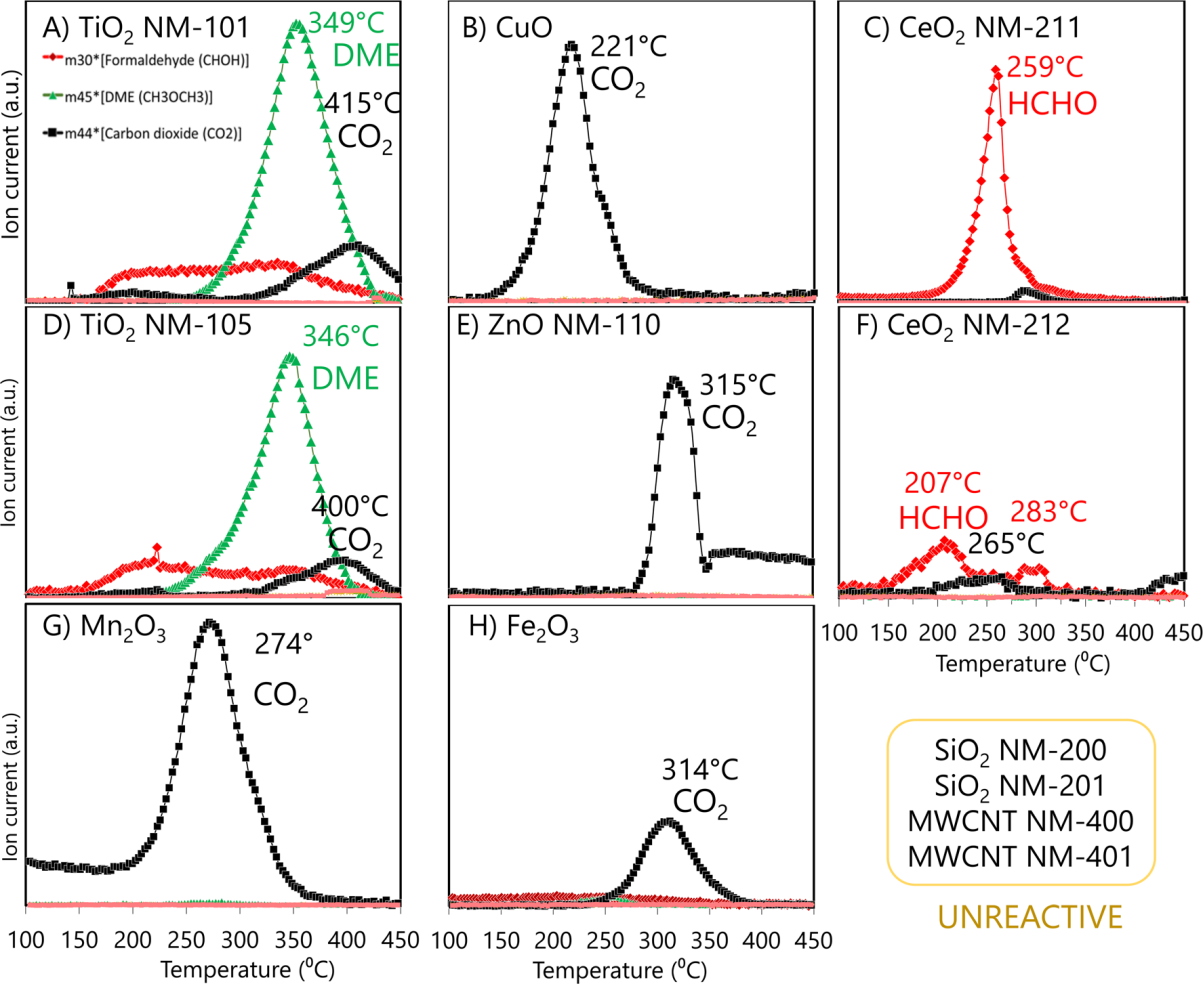
Temperature-programmed surface reaction products of pre-adsorbed methanol analysed by mass spectroscopy of TiO_2_ NM-101 (A), CuO (B), CeO_2_ NM-211 (C), TiO_2_ NM-105 (D), ZnO NM-110 (E), CeO_2_ NM-212 (F), Mn_2_O_3_ (G), and Fe_2_O_3_ (H). Formaldehyde (red diamond) is formed at redox surface sites, dimethyl ether (green triangle) at acid surface sites, and carbon dioxide (black square) at basic or highly reactive redox surface sites. No detectable formation of species with mass 60 (methyl formate) or mass 75 (dimethoxymethane) was observed, indicating the absence of bifunctional reactive surface site activity.

The nature of surface reactivity of the ENMs was tested by MeOH-TPSR (Fig. 1) to identify the type of reactive surface sites and classify them accordingly. TiO_2_ nanomaterials presented a main desorption band related to dimethyl ether formation (Fig. 1A and D), *i.e.*, to acid reactivity, in line with the TPSR results obtained in a previous study with anatase titania DT51 from CristalACTiV™.^26^ In addition, low signals of formaldehyde and carbon dioxide were registered, indicating the presence of some redox and basic sites in titania samples. Ceria, however, exhibited mainly redox reactivity (Fig. 1C and F); NM-211 showed the maximum methanol conversion to formaldehyde at 259 °C, whereas NM-212 formed formaldehyde at two types of sites, more (207 °C) and less (283 °C) active than those of NM-211, with intermediate carbon dioxide generation at around 265 °C. CO_2_ reports two kinds of reactive surface sites: (a) highly oxidising sites readily convert surface methoxy species to CO_2_ with a maximum at temperatures ranging from 170 °C or lower to *ca.* 280 °C; (b) basic sites bind methoxy species so strongly that these may only desorb at high temperature (above 300 °C), being readily converted to CO_2_, as described in the literature.^25^ In the case of ZnO, CuO, Mn_2_O_3_ and Fe_2_O_3_ carbon dioxide was the only reaction product. Fig. 1B shows that CuO was a highly oxidizing material (CO_2_ maximum formation at 221 °C), and Mn_2_O_3_ was the next highly oxidizing material (CO_2_ maximum at 274 °C in Fig. 1G). The results for ZnO and Fe_2_O_3_ are shown in Fig. 1E and H, where it can be observed that CO_2_ formed at significantly higher temperatures, indicating an essentially basic character of the reactive sites. The temperature of maximum CO_2_ formation can be used to rank the reactivity of these four ENMs from a kinetic point of view as follows: CuO (221 °C) > Mn_2_O_3_ (274 °C) > Fe_2_O_3_ (314 °C) ≥ ZnO (315 °C); here, lower temperatures are representative of methanol over-oxidation due to highly reactive redox sites, while methanol combustion is the CO_2_ production mechanism at high temperatures, indicative of strong affinity between basic sites and the methoxy group. Methanol-TPSR data for highly inert SiO_2_ (NM-200 and NM-201) and MWCNT (NM-400 and NM-401) are summarized in Fig. S1.† Only SiO_2_ NM-201 and MWCNT NM-400 showed some reactivity, with a slight band related to CO_2_ formation that suggested a basic character of their low reactivity sites.

### 3.2 Consumption of thiol-based antioxidants

**Fig. 2:**
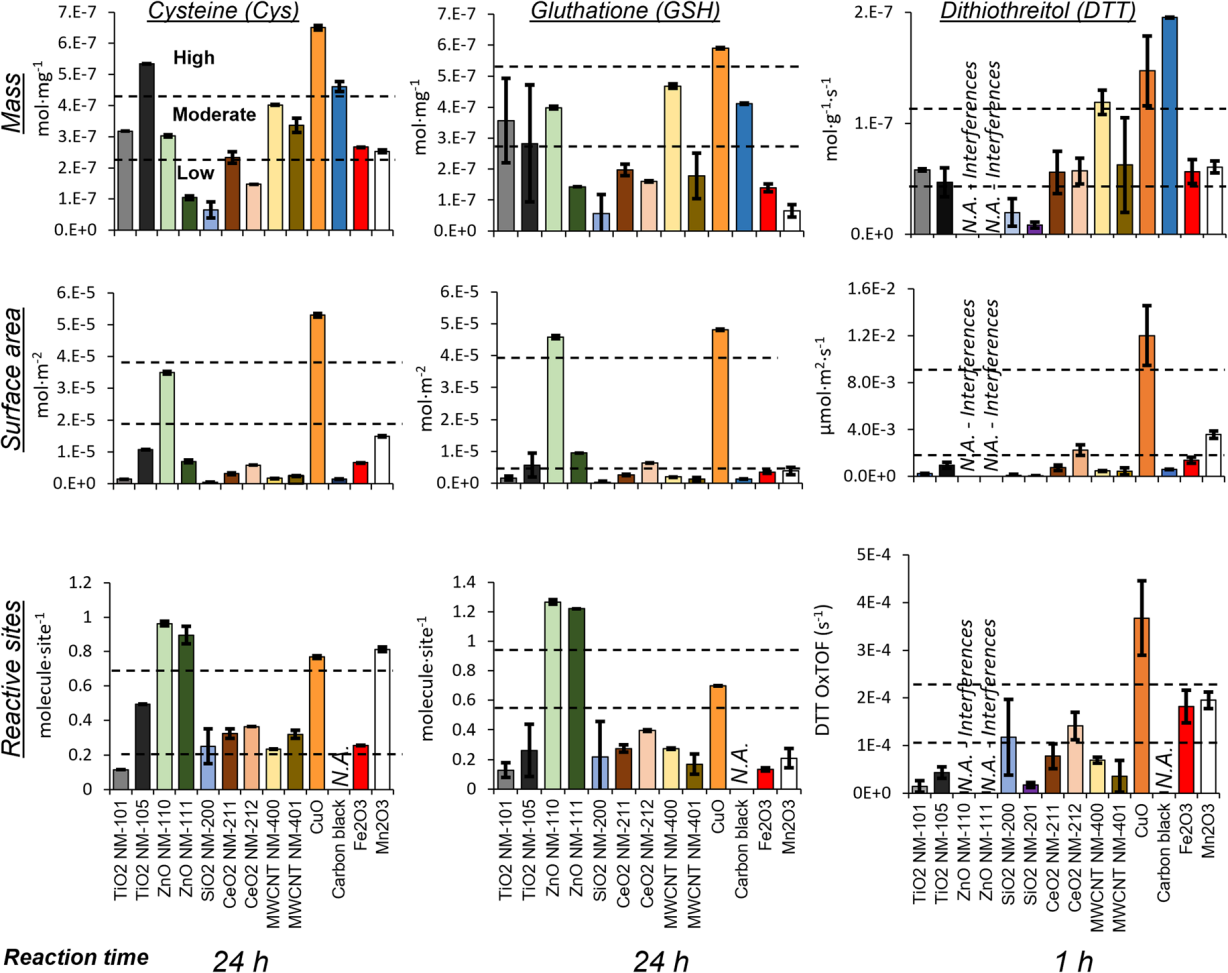
Oxidative potential of the tested ENMs evaluated based on Cys (left) and GSH (center) 24 h consumption and the DTT 1 h oxidation rate (right) normalized by mass (top), surface area (middle) and reactive site (bottom). Averaged values (*n* = 3) with error bars indicating the standard deviation. Statistical clustering by the *k*-means algorithm according to reactivity (high-moderate-low) is indicated by horizontal dashed lines.

The oxidative potential evaluated as Cys and GSH consumption after 24 h and as the DTT oxidation rate after 1 h are summarized in Fig. 2 and sorted by the *k*-means clustering algorithm as high, moderate, or low. SiO_2_ NM-200 was substantially unreactive in all assays, but site normalization indicated a moderate reactivity of its few reactive surface sites for Cys and DTT.

The most active ENM for Cys and GSH consumption was CuO, both per mass and per surface area, whereas active site normalization highlighted the oxidative potential of the zinc oxides, in particular NM-111. Carbon black and TiO_2_ NM-105 consumed quite a large amount of these thiols per unit mass; however, they were in the low reactivity cluster when normalized by surface area or reactive site. Ceria exhibited low thiol consumption in GSH, but moderate Cys consumption per site. MWCNT NM-400 exhibited moderate reactivity for mass-normalized data, but low reactivity according to surface area normalization.

According to DTT consumption assay, carbon black, CuO, and MWCNT NM-400 were classified as highly oxidative according to the mass-based oxidation rate. However, for data normalized per area or reactive surface site, carbon black and MWCNT NM-400 fell into the moderate and low reactivity group, whereas Mn_2_O_3_, Fe_2_O_3_ and CeO_2_ NM-212 were upgraded to highly reactive according to site normalization. Normalization per reactive surface site estimated the low reactivity of TiO_2_ NM-101 and TiO_2_ NM-105, which were, however, clustered as moderately reactive by mass normalization.

### 3.3 Generation of ROS

**Fig. 3:**
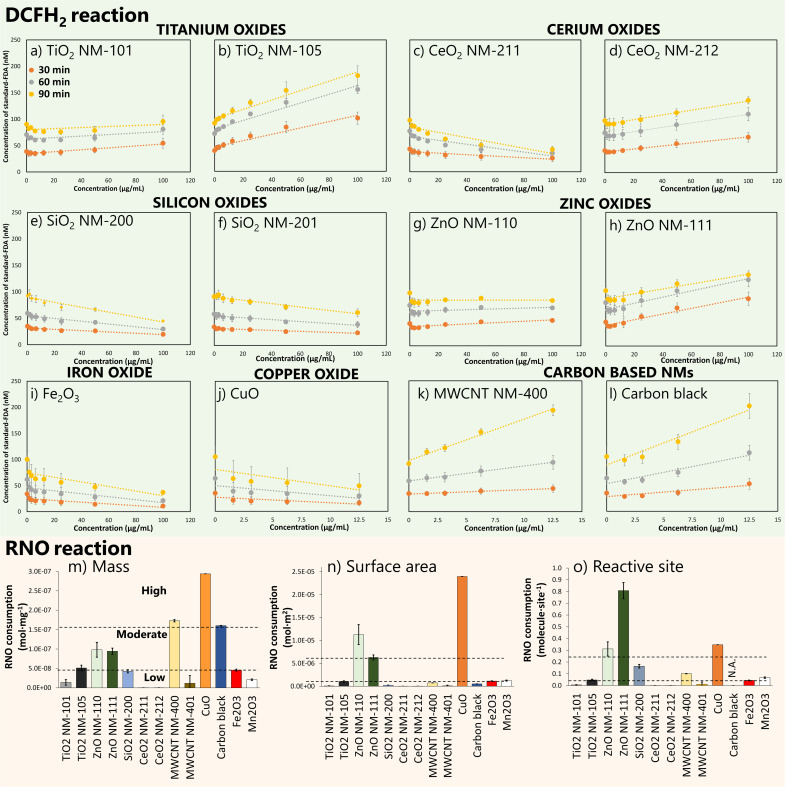
ROS production estimation based on DCFH_2_ assay (a)–(l) and *OH trapping with RNO (m)–(o). Depleted DCFH_2_ at different ENM concentrations measured by using a standard-FDA calibration curve for TiO_2_ NM-101 (a), TiO_2_ NM-105 (b), CeO_2_ NM-211 (c), CeO_2_ NM-212 (d), SiO_2_ NM-200 (e), SiO_2_ NM-201 (f), ZnO NM-110 (g), ZnO NM-111 (h), Fe_2_O_3_ (i), CuO (j), MWCNT NM-400 (k) and carbon black (l). Carbon based NMs and CuO were only tested between 0 and 12.5 μg mL^−1^ according to the results of the interference test. RNO depletion is normalized per mass (m), per surface area (n) and per reactive site (o). Averaged values (*n* = 3) with error bars indicating the standard deviation.

ROS production by nanomaterials was analyzed using DCFH_2_ assay and *OH trapping with RNO. The results of DCFH_2_ assay were expressed as the concentration of standard fluorescein diacetate (FDA) to obtain normalized data. DCFH_2_ depletion (Fig. 3a–l) was observed for carbon black, MWCNT NM-400, ZnO NM-111, CeO_2_ NM-212, TiO_2_ NM-105, and TiO_2_ NM-101, which displayed positive and linear slopes, informing that ROS induction occurred in a dose-dependent manner. The significantly higher slope of NM-105 compared to NM-101 is indicative of higher rates of reactive oxygen species production by NM-105. Unlike CeO_2_ NM-212 and ZnO NM-111, CeO_2_ NM-211 and ZnO NM-110 show no ROS production. Four additional nanomaterials did not show any difference compared to the negative control for ROS: both silicon oxides (NM-200 and NM-201), CuO, and Fe_2_O_3_. Carbon-based ENMs and CuO were only tested between 0 and 12.5 μg mL^−1^ following the interference test results (Table S4†). Thus, DCFH_2_ reaction was unsuitable for detecting ROS generated by CuO.

The results for RNO depletion by *OH trapping are shown in Fig. 3m–o. Five nanomaterials appeared very active in producing *OH: ZnO NM-110, ZnO NM-111, carbon black, CuO and MWCNT NM-400. These data highlight the elevated oxidative potential of zinc oxide reactive surface sites. Low reactivity was observed for TiO_2_ NM-105, Fe_2_O_3_ and Mn_2_O_3_, while TiO_2_ NM-101, both CeO_2_ and MWCNT NM-401 performed similarly to the negative control. A large standard deviation for MWCNT NM-401 data was caused by non-stable dispersions in water, probably due to its high hydrophobicity.

## 4 Discussion

### 4.1 ENM's reactive surface sites

The reactive site surface density of the tested ENMs in this work ranged between 0.8 and 22 sites per nm^2^ (Table 1), consistent with literature data.^23,26,36–39^ Methanol chemisorption was also performed at 50 °C for highly reactive CuO and ZnO NM-110 materials to avoid the formation of methoxy multilayers due to strong reactive interactions. The reactive site surface densities obtained at 100 °C were higher than those corresponding to a monolayer, which is in line with the observed CO_2_ formation in MeOH-TPSR *via* decomposition of carbonates (high temperatures) and formates (low temperatures).^40–42^ The carbonates form at basic sites, while formates originate at redox sites. SiO_2_ NM-200 and NM-201 presented a low specific number of reactive sites, also resulting in low reactive sites surface density; these results explained the low reactivity observed in all assays and the absence of MeOH-TPSR products. We conclude that, although very large, the surface of these silica ENMs was mostly unreactive. The same behavior was observed for carbon nanotubes (MWCNT NM-400 and MWCNT NM-401). Despite their high specific surface area, they were actually two of the engineered nanomaterials with the lowest reactive site surface density, only slightly higher than that of silica oxides. Therefore, due to their low specific number of reactive sites and the absence of products in TPSR-MeOH, it is suggested that the reactivity observed for NM-400 in the liquid-phase reactions must be produced by reactive contaminants (Table S1†).^43,44^

The chemisorption capacity of ZnO NM-111 seemed to be limited by the triethoxycaprylsilane coating, so that only about 5 reactive sites per nm^2^ were available to interact with methanol, whereas the uncoated ZnO NM-110 showed a smaller area but a very high surface density of reactive sites. Their reactive surface sites were similarly high consumers of GSH and Cys antioxidants in both nanomaterials. The site-normalized reactivity highlighted the oxidative capacity of NM-110 and NM-111 in Cys and GSH consumption tests.

### 4.2 Nanomaterial ranking

**Fig. 4:**
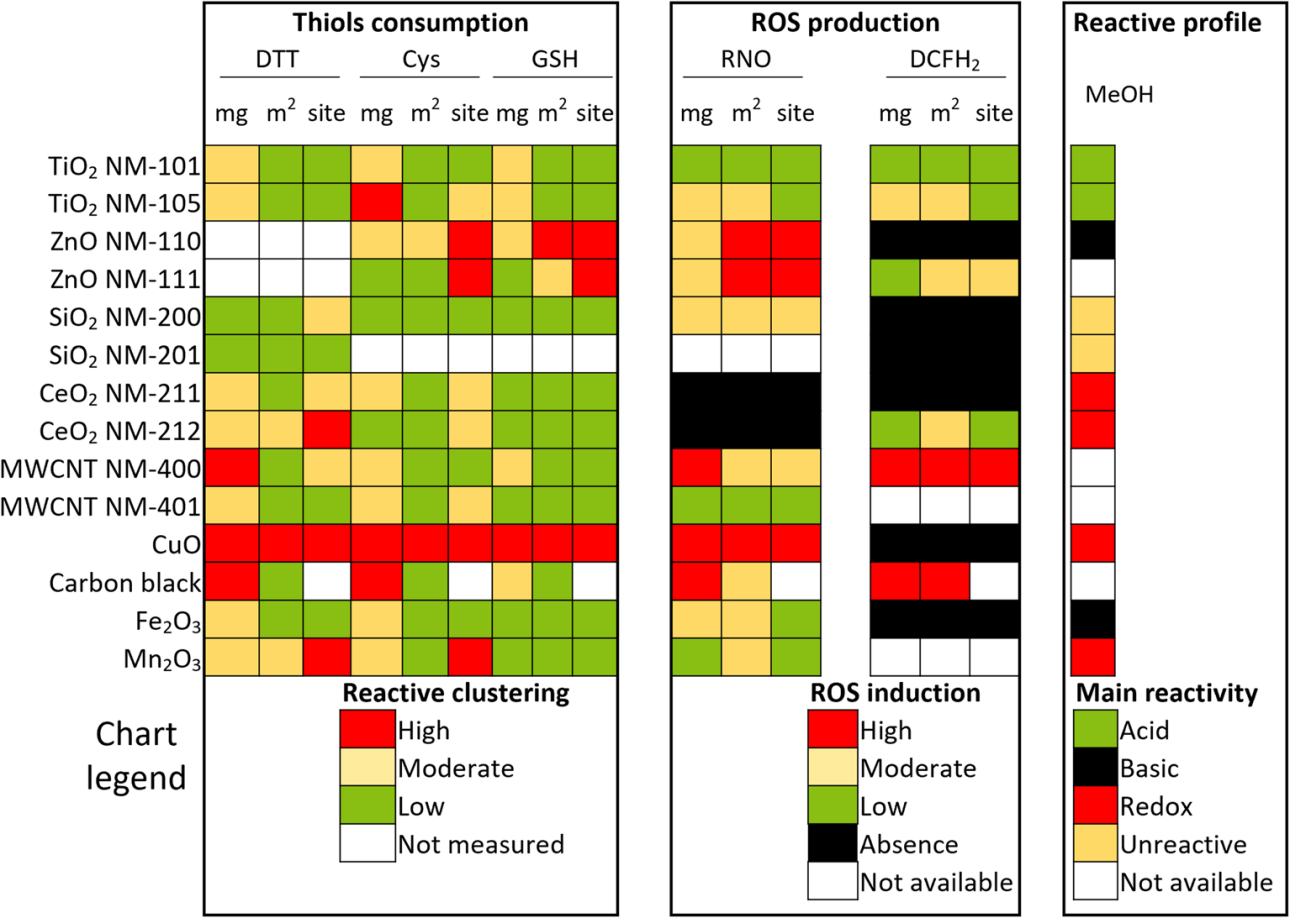
Heat map for the 14 ENMs evaluated based on their intrinsic oxidative capacity to react with the thiol group in DTT, Cys, and GSH, or their production of ROS that are trapped by RNO and DCFH_2_, as well as based on their reactive profile obtained *via* methanol temperature programmed surface reaction. Clustering was performed by the *k*-means algorithm.

To compare, rank and categorize the ENMs by reactivity, we adopted the *K*-means clustering algorithm (Fig. 4). CuO stands out as the ENM with the highest oxidative potential, as it was consistently classified as highly reactive across all antioxidant probe molecules, including *OH radicals observed in RNO depletion. However, in the DCFH_2_ probe reaction, the interference caused by this ENM prevented the detection of ROS. Following CuO, the subsequent nanomaterials in the reactivity scale were the two ZnO (NM-111 and NM-110) and carbon black. CuO and uncoated ZnO (NM-110) showed great similarities: they exhibited a high oxidizing character, form CO_2_ in MeOH-TPSR, but did not react against DCFH_2_; in addition, their consumption of RNO suggest that they form *HO.

Reported ESR and FRAS (ferric reducing ability of the serum) assay data for ZnO NM-110 ^17,18^ confirm the absence of ROS production. Moreover, a high depletion of cyclic hydroxylamine spin probe 1-hydroxy-3-carboxy-pyrrolidine (CPH) and nitrone spin trap 5,5-dimethyl-1-pyrroline-*N*-oxide (DMPO) molecules, sensitive to different ROS, was reported for this material compared to the blank, and surface normalized data provided further evidence of the oxidative capacity.^19,35^ The FRAS assay revealed high oxidation levels caused by ZnO NM-110, in line with its reactivity per site in GSH, Cys, and RNO reactions. Similar to this uncoated ZnO, silane coated ZnO (NM-111), one of the most oxidizing ENMs in the series, tested positive in FRAS experiments,^35^ as well as CPH and DMPO probes.^19^ The different behavior of NM-110 *vs.* NM-111 in the DCFH_2_ probe reaction suggests that both nanomaterials have different oxidation mechanisms, being NM-111 a nanomaterial with higher ROS induction capacity.

Lastly, carbon black was utilized as a positive control in DCFH_2_ assay due to its oxidative capacity evidenced by the high ROS production, high DTT depletion and high sensitivity for FRAS assay reported in the literature.^15,45–48^ This high reactivity of CB agreed with the results obtained in this study by mass normalization, where a high consumption of GSH, Cys, DTT, and RNO, and especially a high slope in the DCFH_2_ reaction were obtained, according to its capacity to induce ROS.

These data clustered SiO_2_ NM-200, Fe_2_O_3_, CeO_2_ NM-211, CeO_2_ NM-212, TiO_2_ NM-101 and TiO_2_ NM-105 in a lower reactivity group. It is worth noting the similarity between the two titanias reflected by MeOH-TPSR, mainly when compared to the higher oxidizing capacity of ceria NM-211 *vs.* NM-212. The different oxidative potential for CeO_2_ ENMs is supported by XPS results in the literature, which detected a higher percentage of Ce(III) on the surface of NM-211 (22%) *vs.* NM-212 (14%).^49^ Concerning SiO_2_ NM-200, it exhibited low reactivity in all reactions and metrics, except for DTT consumption per active site. The limited number of reactive sites on the surface leads to an artificially high value of molecule consumption per site, even though the nanomaterial is, in fact, classified as unreactive. There is no substantial consumption of any molecule, no measurable ROS formation, and no products observed in MeOH-TPSR whatsoever. This evidence underlines the relevance of the binomial constituted by (a) the number of reactive surface sites and (b) the reactivity of such sites.

The results of oxidative potential per reactive surface site obtained with the different probe molecules were compared utilizing Pearson's correlation coefficient (Fig. S2†). The comparison of probe molecules enabled an exploration of the underlying reaction mechanisms governing their consumption. The significant Pearson correlation coefficient between GSH, Cys, DTT and RNO (0.91–0.80) suggests a shared oxidation mechanism. As outlined in previous studies,^50–52^ this mechanism primarily involves the oxidation of thiol (–SH) groups, which are critical functional components in GSH, Cys, and DTT. During this process, thiols undergo a single-electron oxidation, resulting in the formation of sulfur-centered radicals that later combine to form disulfides. Other authors, such as Jiang and colleagues, attributed the oxidation of DTT to the presence of transition metals, like Cu, which induce Fenton-like reactions, producing H_2_O_2_ and ultimately generating *OH radicals.^53^ Consequently, for ENMs like CuO, the consistent data showing oxidation of –SH groups and generation of *OH radicals that deplete RNO are in perfect agreement. Additionally, the surface of CuO particles is predominantly reduced to either Cu_2_O or metallic copper, as demonstrated by Yiwen Wang *et al.*^54^ For ZnO and TiO_2_, thiol adsorption has been reported in the literature; however, the acidic surface of TiO_2_ may not oxidize DTT, while the highly reactive surface sites of ZnO result in significant depletion of –SH antioxidants. In contrast, CeO_2_, with its oxidative surface sites, exhibits only slight oxidation of antioxidants, likely due to the absence of –SH adsorption on its surface.^55^ However, DCFH_2_, which theoretically should provide results similar to RNO, does not correlate with the obtained dataset for ROS generation (RNO) and antioxidant consumption (DTT, Cys, and GSH). This discrepancy is primarily attributed to interference from carbon-based and CuO ENMs, while showing no reactivity in the presence of Zn^2+^, as evidenced by the low reactivity of ZnO NM-110.

### 4.3 Correlation between surface reactivity and *in vitro* toxicity data: a new dosimetry approach

The results for *in chemico* reactivity described in this work and literature cytotoxicity data suggest that enhanced reactivity in the tested ENMs correlates with increased cytotoxicity in pulmonary cells post 24 h exposure. Notably, CuO, ZnO NM-110, and ZnO NM-111, identified as highly oxidizing, markedly reduced cell viability in primary pulmonary cell lines. Specifically, CuO emerged as the most cytotoxic ENM, significantly impacting A549 cell viability at concentrations of 5–10 μg mL^−1^ across MTT, NRU, CFA, and IL-8 assays,^56–59^ while zinc oxide NM-110 exhibited significant effects on cell viability at 6.4–50 μg mL^−1^ for PMA treated THP-1,^60,61^ 128 μg mL^−1^ for Calu-3 ^62^ and 48–75 μg mL^−1^ for A549,^63–65^ and even lower concentrations are required for ZnO NM-111: 37 μg mL^−1^ in A549 ^65^ and 32–128 μg mL^−1^ in Calu-3.^62^ Exposure to CuO, ZnO NM-110 and ZnO NM-111 may result in different modes of action, including those derived from ion release into the medium, as they are known to dissolve. For example, in a recent *in vivo* study in rats, ZnO NM-110 was compared to highly soluble ZnSO_4_, concluding that ZnO nanoforms most likely exhibit their effects by zinc ions, since the exposure to zinc sulfate had similar effects. However, they are highly reactive not only in the consumption of probe molecules during dissolution, but also in methanol-TPSR, which is performed with the powdered material, suggesting that surface reactivity may provide very relevant complementary information for toxicity evaluation.^66^ Indeed, it is remarkable that Cronholm *et al.* observed significant cell and DNA damage caused by CuO nanoparticles but no adverse effects from highly soluble CuCl_2_ salts.^67^ In agreement with this, Wang *et al.* concluded that dissolved Cu^2+^ ions contributed to less than half of the total effects caused by CuO NPs, including ROS generation and DNA damage.^68^ Thus, we identify surface reactivity as one of the key factors driving the electronic imbalance within cells, ultimately resulting in cellular damage.

Exposure to CuO, ZnO NM-110 and ZnO NM-111 may result in different modes of action. In contrast, exposure to non-soluble ENMs with moderate or low oxidative potential causes less pronounced effects in pulmonary cells. TiO_2_ NM-101 produces no significant alteration in BEAS-2B and A549 cell viability.^69–71^ Similarly, TiO_2_ NM-105 shows no notable impact on A549 and THP-1 cell lines.^72,73^ Cerium oxide NM-212 does not disrupt LDH, cytokine release, or Alamar blue assay markers in A549 and PMA-treated THP-1 cells.^73,74^ SiO_2_ NM-200 cytotoxicy has been tested for BEAS-2B,^70^ Calu-3 ^62^ and PMA treated THP-1,^60^ but significant effects were on;y obtained with the MTS test on THP-1 at a concentration of 50 μg mL^−1^. An MTT assay in A549 and BEAS-2B indicates no impact on cell viability after 24 h exposure to 200 μg mL^−1^ Fe_2_O_3_.^75^ Conversely, carbonaceous materials exhibit varied effects. Exposure of A549 cells to 50 μg mL^−1^ carbon black induces ROS and IL-8 release without affecting cell viability, whereas no cytotoxicity is observed in Calu-3 at this concentration.^76^ MWCNT NM-400 at 100 μg mL^−1^ in A549 prompts LDH release and reduces cell viability, and at 80 μg mL^−1^ in PMA-treated THP-1 it triggers IL-8 release. MWCNT NM-400 at 100 μg mL^−1^ in A549 induced LDH release and decreased cell viability,^77^ and at 80 μg mL^−1^ in PMA treated THP-1 it triggers IL-8 release.^78^ The toxicity of MWCNT NM-400 produced by surface reactivity could be explained due to their impurities;^44^ however, it is not expected to initiate reactions leading to toxicity effects.^43,77,79^ Additionally, based on previous studies, these impurities are not released in the medium.^79^ Finally, when MWCNT NM-401 is exposed to PMA treated THP-1 and A549, the main effect observed is IL-8 release at 40 μg mL^−1^ for both cell lines.^78^

Understanding the interplay between toxicity and reactivity poses a complex challenge, particularly without complementary assays addressing biotransformation, bioaccumulation, corona protein formation, and related factors. Moreover, the variety of reactivity and toxicity testing methods and conditions employed in different studies hampers a comprehensive analysis. Taking this into account, available toxicity data of TiO_2_ NM-101, TiO_2_ NM-105, ZnO NM-110, ZnO NM-111, SiO_2_ NM-200, SiO_2_ NM-201, CeO_2_ NM-211, CeO_2_ NM-212, CuO and Fe_2_O_3_ evaluated by LDH and WST-1 assays with A549 and dTHP-1 cell lines, collected in Table S3,† were selected owing to their relative uniformity (same two assays and two cell lines), same exposure time (24 h) and the extensive range of tested ENMs (11 out of the 14 studied ENMs).^80^ These data are expressed as no observed adverse effect concentration (NOAEC) of reactive sites concentration (μmol L^−1^), our new proposed dose metric, to calculate Spearman's coefficients summarized in Table 2, aiming to evaluate possible correlations between oxidative reactivity against the five specific probe molecules used in this work and the reported cell viability. DCFH_2_ depletion rates were measured at 50 μg mL^−1^ of ENMs, and normalized by mass, area and sites (Table S2†). Oxidative reactivity in cell-free environments and *in vitro* toxicity values exhibit negative Spearman's coefficients, indicating an inverse proportionality. This means that ENMs with higher reactivity require lower concentrations to induce cell damage. DTT, GSH, Cys and RNO reactions showed similar moderate Spearman correlation factors for *in vitro* adverse effects; the least predictive marker is the oxidation rate of DCFH_2_, which demonstrates a lower prognostic efficacy for A549 and dTHP-1 cell viability.

**Table 2:** Spearman correlation between oxidative potential per reactive surface site measured by DCFH_2_, RNO, DTT, Cys and GSH probe reactions in this work and bibliographic *in vitro* toxicity data assessed by LDH and WST-1 tests in A549 and dTHP-1 cell lines after 24 h exposure from Table S3. Raw toxicological data extracted from Alcolea-Rodriguez *et al.*^80^

		RNO	GSH	Cys	DTT	DCFH_2_
A549	LDH	−0.55	−0.54	−0.56	−0.53	−0.16
A549	WST-1	−0.57	−0.58	−0.59	−0.53	−0.21
dTHP-1	LDH	−0.55	−0.54	−0.56	−0.53	−0.16
dTHP-1	WST1	−0.57	−0.59	−0.59	−0.53	−0.21
	*n*	9	9	9	8	9

This new dosimetry shares the limitations of other normalizations when applied to soluble nanomaterials: ion secretion results in mass loss (leading to a reduction in nanomaterial concentration) and surface area alteration (with the exposed surface area remaining unknown). Consequently, if ions originating from the ENM are dissolved in the reaction media, in addition to interference problems, it is not possible to ascertain the state of reactive sites. Therefore, for cytotoxic ENMs such as CuO and ZnO (NM-110 and N-111) that are partially soluble, it is challenging to determine their true exposure levels based on reactive surface site concentrations. Other nanomaterials not exhibiting solubility in the literature, such as titania, Fe_2_O_3_, and MWCNTs,^79^ exhibit significant disparities in their actual exposure of reactive sites for a similar concentration, *e.g.*, 100 μg mL^−1^ of TiO_2_ NM-101 presents a high number of reactive sites (280 μmol sites per L) compared to its analog NM-105 (110 μmol sites per L) or to other nanomaterials like Fe_2_O_3_ (110 μmol sites per L). This phenomenon highlights the relevance of using reactive site concentration as an alternative to gravimetric concentration, as comparisons in terms of mass that do not account for the exposure of each nanomaterial. However, when using volumetric surface area (cm^2^ mL^−1^), the Spearman correlation factors for GSH, Cys, and DTT depletion increase to approximately 0.80 (data not shown) compared to cell-based parameters, indicating a stronger correlation. This trend arises because the most reactive ENMs, ZnO and CuO, have low BET surface areas. We expect that, since not all physical areas correspond to chemical areas, expanding the study to include a broader cohort of more spherical ENMs will likely reduce the Spearman correlation. The current high correlation is due to the limited number of cytotoxic ENMs in this study.

## 5 Conclusions and outlook

This study reinforces the significance of reactivity by introducing new probe-molecule methods to evaluate reactivity that produce results comparable with established assays, such as DCFH_2_. Our measurements aimed to establish that (1) methods such as methanol-TPSR, antioxidant consumption, and ROS generation measured by RNO and DCFH_2_ can elucidate the surface reactivity of ENMs; (2) ENMs with identical compositions can exhibit varying oxidative capacities; (3) the number and nature of reactive surface sites significantly influence their reactivity and, consequently, their toxicity; and (4) a site-based dose metric is proposed for establishing the correct effective dose of non-soluble ENMs.

The reactivity screening conducted in this study with 14 ENMs using 6 probe reactions underscores the oxidizing potential of nanomaterials such as CuO, ZnO NM-110, ZnO NM-111, and carbon black, whereas nanomaterials such as MWCNT NM-400, TiO_2_ NM-105, CeO_2_ NM-212, SiO_2_ NM-200, TiO_2_ NM-101, CeO_2_ NM-211, MWCNT NM-401, Mn_2_O_3_, and Fe_2_O_3_ are categorized into groups with moderate to low oxidative reactivity. Additionally, distinct oxidative mechanisms were proposed for each ENM. For ZnO, its reactivity and toxicity have been reported in the literature as dependent on the release of Zn^2+^ ions. In contrast, CuO's surface reactivity emerged as the primary mechanism of oxidation and toxicity, as Cu salts, in comparison, failed to induce similar toxicity or reactivity effects according to the literature. These findings reflect the complex mechanisms ocurring at the nanolevel, and highlight the multifactorial mode of action of ENMs in biological systems.

The proposed methodology offers a deep understanding of the reactivity of ENMs. It has versatile applications, among which a novel approach is suggested for normalizing toxicity data. This method considers the reactive surface sites rather than solely relying on the mass or the total surface area of ENMs. Such an approach could significantly enhance the field of nanotoxicology in the future, providing a more accurate risk assessment of ENMs.

## Data availability

The data supporting this article have been included as part of the ESI.†

## Conflicts of interest

The authors declare no competing financial interest.

## Supplementary Material

NA-007-D5NA00104H-s001
