# Supplementary material for: A refined dose metric for nanotoxicology based on surface site reactivity for oxidative potential of engineered nanomaterials
Source: Nanoscale Adv. 2025 Feb 26;7(10):2929–41. doi: 10.1039/d5na00104h (PMC11956195; doi:10.1039/d5na00104h)
Supplement: NA-007-D5NA00104H-s001 [file NA-007-D5NA00104H-s001.pdf]

Supplementary Information (SI) for Nanoscale Advances.
This journal is © The Royal Society of Chemistry 2025

A refined dose metrics for nanotoxicology based on surface sites reactivity for oxidative potential of engineered nanomaterials

Victor Alcolea-Rodriguez[a,b*], Felice C. Simeone[c], Nicolás Coca-López[a], Verónica I. Dumit[b], Lara Faccani[c], Andrea Haase[b], Raquel Portela[a], Miguel A. Bañares[a*]

[a] CSIC-ICP, Instituto de Catálisis y Petroleoquímica, Spectroscopy and Industrial Catalysis (SpeiCat), Marie Curie 2, 28034-Madrid, Spain.

[b] Department of Chemical and Product Safety, German Federal Institute for Risk Assessment, Berlin 10589, Germany

[c] National Research Council of Italy (CNR), Istituto per la Scienza, Sostenibilità e Tecnologia dei Materiali Ceramici (ISSMC), Via Granarolo, 64, 48018 Faenza (RA), Italy

***Corresponding author**: Miguel A. Bañares

Table of content

1 Nanomaterials ... 2
2 Reactive characterization methods ... 3
2.1 Consumption of DTT anitoxidant ... 3
2.2 Consumption of glutathione and cysteine antioxidants ... 3
2.3 Hydroxyl radicals trapping with RNO ... 3
2.4 $DCFH_2$ assay for ROS detection ... 3
3 Results ... 5
3.1 $DCFH_2$ depletion ... 5
3.2 Methanol-TPSR ... 5
3.3 Probe molecules correlation ... 6
3.4 Reactive site-based dose-metrics applied to *in vitro* toxicity data ... 6
3.5 Probe molecules and normalization methods: comparison ... 6
3.6 Raw data of DTT, Cys, GSH and RNO reactions ... 1
4 REFERENCES ... 1
5 ACKNOWLEDGMENTS ... 3

1 Nanomaterials

Table S 1. *Physicochemical properties reported in the literature for the studied nanomaterials from JRC, Sigma-Aldrich and PRINTEX.*

NM	Supplier code	Particle size (nm)	Particle size distribution (nm)	Specific surface area (m^2/g)	Other information	Ref.
**TiO_2 NM 101**	JRCNM01001a	5-9	<100 → 95%<br><50 → 77 %<br><10 → 11 %	170/316	Anatase<br>Thermal synthesis<br>Photocatalytic activity	1–3
**TiO_2 NM 105**	JRCNM01005a	15-24	NA	52.81-55.49<br>46	83% anatase-17% rutile	1,2
**CeO_2 NM 211**	JRCNM02101a	10.3	In water:<br>D_{10} 810 ± 160<br>D_{50} 202 ± 17<br>D_{90} 130 ± 60	65	Precipitated<br>Ce:O = 1:2 (XPS)	2,4
**CeO_2 NM212**	JRCNM02102a	28.4 ± 10.4	NA	27.2 ± 0.9	Cubic cerionite	2,4
**ZnO NM 110**	JRCNM62101a	202.6 ± 12.8	DI water (nm)<br>D10 286±2<br>D50 82.8±1.9<br>D90 107.3±1.7	12.4	Zincite<br>ζ: -19.1 ± 0.5 mV	2,5
**ZnO NM 111**	JRCNM01101a	140.8 ± 65.8	NA	15.1 ± 0.6	Coated with triethoxycapryl silane	2,5
**SiO_2 NM 200**	JRCNM02000a	14-23	<100 → 89%<br><50 → 70 %<br><10 → 2 %	189	Amorphous<br>Precipitated	2,6
**SiO_2 NM 201**	JRCNM02001a	17-20	<100 nm - 81.5%,<br><50 nm – 55.3%<br><10 nm – 1.1%	140	ζ (pH 6.9, milliQ water): -51.7 mV<br>Na-4400 ppm, Al- 7400 ppm, S- 4600 ppm, Si -45.27 wt %, O- 53.08 wt%	2,6
**Fe_2O_3**	544884 (Sigma-Aldrich)	35 ± 14	NA	39	Impurities <0.5%	7
**CuO**	544868 (Sigma-Aldrich)	33.3 ± 10.7	10-100 nm	11	Tenorite<br>77-82.6 % Cu<br>Crystallite size: 17 nm<br>IEP: 8.3, ζ: 15.1 ± 9.4 mV	8–11
**Mn_2O_3**	?	?	?	?	?	
**Carbon black**	Printex90	Particle: 19<br>Aggregate: 58	?	317	ellipsoidal primary particles aspect ratio of 1,4 – 1,5	12
**MWCNTs NM400**	JRCNM04000a	L: 846<br>D: 11	-	254	-	2,13–15
**MWCNTs NM401**	JRCNM04001a	L:4048<br>D: 67	-	140	-	2,13–15

2 Reactive characterization methods

2.1 Consumption of DTT anitoxidant

A 200 µg/mL suspension of ENM in 1 mM phosphate buffer is prepared by sonication, following NanoGenoTox SOP (16 min at 400 W and 10% amplitude). A total of 3 mL of the ENM suspension is incubated for 1 h at 37 °C and 500 rpm with 3 mL of 100 µM DTT, following a procedure described elsewhere (Alcolea-Rodriguez et al, under revision) DTT oxidation rate was normalized per mass ($mol·g^{-1}·s^{-1}$), surface area ($µmol·m^{-2}·s^{-1}$) or reactive surface sites, that is DTT OxTOF (s^{-1}).

2.2 Consumption of glutathione and cysteine antioxidants

A 1 mM suspension of ENM in 2 mM solution of GSH and Cys in PBS (0.01 M; pH=7.4) was prepared in a 12 mL test tube. After incubation for 24 h, the suspension was filtered to eliminate the nanoparticles. Non-oxidized thiol groups were quantified by adding the Ellman reagent up to a final concentration of 3 mM. Absorbance at 410 nm was measured to calculate the amount of unreacted -SH groups from a calibration curve. For each ENM, -SH consumption was measured in triplicate.

2.3 Hydroxyl radicals trapping with RNO

A 2 mM ENM suspension in PBS was mixed in a test tube with 3 mM RNO. After 24 h incubation, nanoparticles were filtered out and the absorbance of the solution at 430 nm was measured to calculate RNO depletion from a linear calibration curve, and thus quantify the production of OH* radicals. The assay was repeated in triplicate for each ENM.

2.4 $DCFH_2$ assay for ROS detection

$DCFH_2$-DA is a lipophilic non-fluorescent probe molecule commonly used in a well-known methodology for NMs screening in previous literature[16,17,71 18, 19, 20] to unravel ROS induction in both a cell-free environment and *in vitro* assays. The reactive mechanism is based on a first step of diacetate removal by alkaline hydrolysis, followed by the reaction between different kinds of generated ROS species and $DCFH_2$ to produce DCF^-, which is quantified by fluorescence. $DCFH_2$-DA was hydrolyzed to $DCFH_2$ by incubation of 0.01M NaOH with 200 µM $DCFH_2$-DA at room temperature for 30 min in a covered vessel protected from light. Then, 0.1 M PBS was introduced to finish deacetylation, until a concentration of 50 µM $DCFH_2$. A second dilution was performed with 0.01M PBS to obtain a 10 µM solution that

was kept on ice until use. Test ENM stock suspensions were prepared by sonication following NanoGenoTox SOP at a concentration of 2.56 mg/mL. Then, dilutions to 15, 31, 63, 125, 250, 500 and 1000 µg/mL were prepared with phenol red-free Minimum Essential Medium (MEM). Finally, using a multi-channel pipette, in each of the wells of a clear-flat-bottom 96-well plate 225 µL $DCFH_2$ solution was added to 25 µL ENM suspension. Final test ENM concentration in reaction mixtures was: 1.5, 3.1, 6.3, 12.5, 25.0, 50.0 and 100.0 µg/mL. Fluorescence at ex/em 485/520 was measured immediately after reaction sample preparation as well as after 30-, 60- and 90-min incubation performed at 37 ºC protected from light. The assay was done per triplicate (n=3). Nanomaterial interference was tested employing positive controls and a calibration curve to ensure the proper execution of the assay.

Calibration. Fluorescein solutions were used as standard to obtain a calibration curve for quantification. Fluorescein diacetate (F-DA) 200 µM was incubated with 0.01M NaOH at room temperature, protected from light, for 5 min. The reaction finished when 0.1M PBS was added, generating fluorescein 50 µM. 0.01M PBS was used to prepare serial dilutions at the following concentrations: 0.001, 0.004, 0.012, 0.037, 0.111, 0.333, 1.000 µM.

Positive controls. Printex® 90 carbon black and 3-Morpholinosydnonimine hydrochloride (SIN-1) were used as nanomaterial and chemical positive controls, respectively. Suspensions were prepared following the same procedure as with the test ENMs for final concentrations of 1.6, 3.1, 6.3 and 12.5 µg/mL. SIN-1 100 µM solutions were prepared in MEM and diluted to 13, 25 and 50 µM to provide a final assay concentration after mixture with $DCFH_2$ of 1.3, 2.5, 5.0 and 10.0 µM.

Nanomaterial interference evaluation. Fluorescence quenching and auto-fluorescence of ENMs was assessed at all experimental doses (from 1.6 to 100 µg/mL) in a black clear-bottom 96-well plate. 225 µL of F-DA or PBS were added to 25 µL of test ENM dispersion to evaluate fluorescence or auto-fluorescence quenching, respectively. All wells were immediately measured at ex/em 485/520.

3 Results

3.1 DCFH$_2$ depletion

Table S 2. *DCFH$_2$ depletion rates at 50 µg/mL of ENM normalized by mass, area and sites. ENMs were clustered using k-means algorithm into three reactivity categories for ROS generation: high, moderate or low.*

	Mass		Area		Site (OxTOF)	
Nanomaterial	Reaction rate (nmol·s^{-1}·g^{-1})	k-means cluster	Reaction rate (µmol·s^{-1}·m^{-2})	k-means cluster	Reaction rate (s^{-1})	k-means cluster
**TiO_2 NM-101**	0.02	Low	$4.81·10^{-8}$	Low	$3.9·10^{-9}$	Low
**TiO_2 NM-105**	0.33	Moderate	$5.77·10^{-6}$	Moderate	$3.0·10^{-7}$	Low
**ZnO NM-111**	0.07	Low	$4.76·10^{-6}$	Moderate	$7.1·10^{-7}$	Moderate
**CeO_2 NM-212**	0.10	Low	$3.94·10^{-6}$	Moderate	$2.5·10^{-7}$	Low
**MWCNT NM-400**	2.46	High	$1.03·10^{-5}$	High	$1.4·10^{-6}$	High
**Carbon black**	2.77	High	$8.73·10^{-6}$	High	NA	NA

3.2 Methanol-TPSR

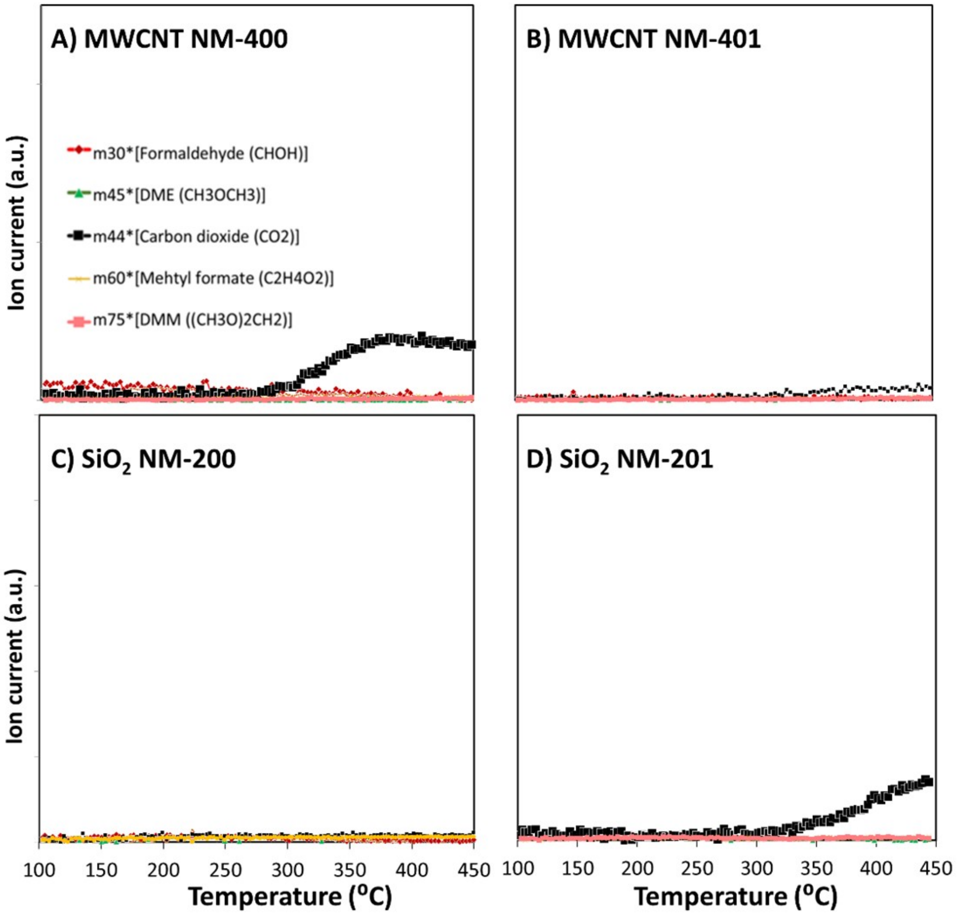

Figure S 1. *Temperature-programmed surface reaction products of pre-adsorbed methanol analysed by mass spectroscopy for two different MWCNT (NM-400 (a) and NM-401 (b),and two different SiO$_2$ (NM-200 (c) and NM-201 (d)). Formaldehyde (red) is formed at redox sites, dimethyl ether (green) at acid sites, and carbon dioxide (black) at basic or high reactive redox sites.*

3.3 Reactive site-based dose-metrics applied to *in vitro* toxicity data

Table S 3. *No observed adverse effect concentration (NOAEC) of reactive sites (µmol / L) for in vitro assays with WST-1 and LDH release tests performed in A549 and dTHP-1 cell lines after 24 h exposure to ENMs. Data source: Alcolea-Rodriguez et al*[21]

	A549		dTHP-1	
**ENM**	**LDH**	**WST-1**	**LDH**	**WST-1**
**TiO_2 NM-101**	280	280	280	280
**TiO_2 NM-105**	110	110	110	110
**ZnO NM-110**	20	2.5	20	0.28
**ZnO NM-111**	5	1.25	10	0.3
**SiO_2 NM-200**	30	30	30	30
**SiO_2 NM-201**	50	50	50	50
**CeO_2 NM-211**	40	40	40	40
**CeO_2 NM-212**	40	40	40	40
**CuO**	1.2	1.2	1.2	2.4
**Fe_2O_3**	100	100	100	100

3.4 Probe molecules and normalization methods: comparison

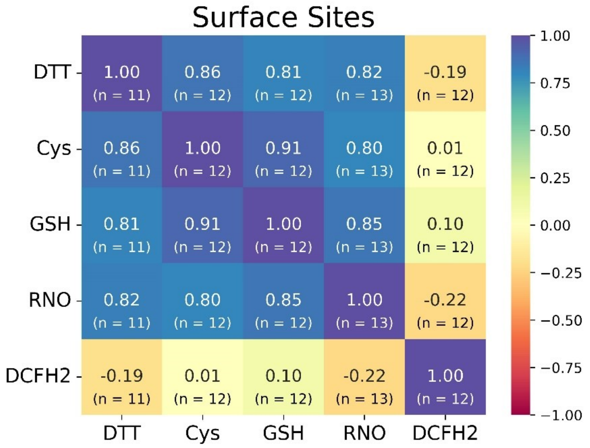

Figure S2*. Heatmap of Pearson's correlation coefficients for the probe reaction results normalized by reactive site.*

The pairplot in *Figure S3* supports our hypothesis, due to it emphasizes the pairwise relationships among the normalization strategies applied to the probe reactions under study, indicating that depending on the probe molecule the data tend to cluster into three distinct groups. The first group comprises Cys, GSH, and RNO; the second group consists of DTT,

and the third group comprises $DCFH_2$. Within each of these groups, the relationships among the normalization techniques approximate linearity.

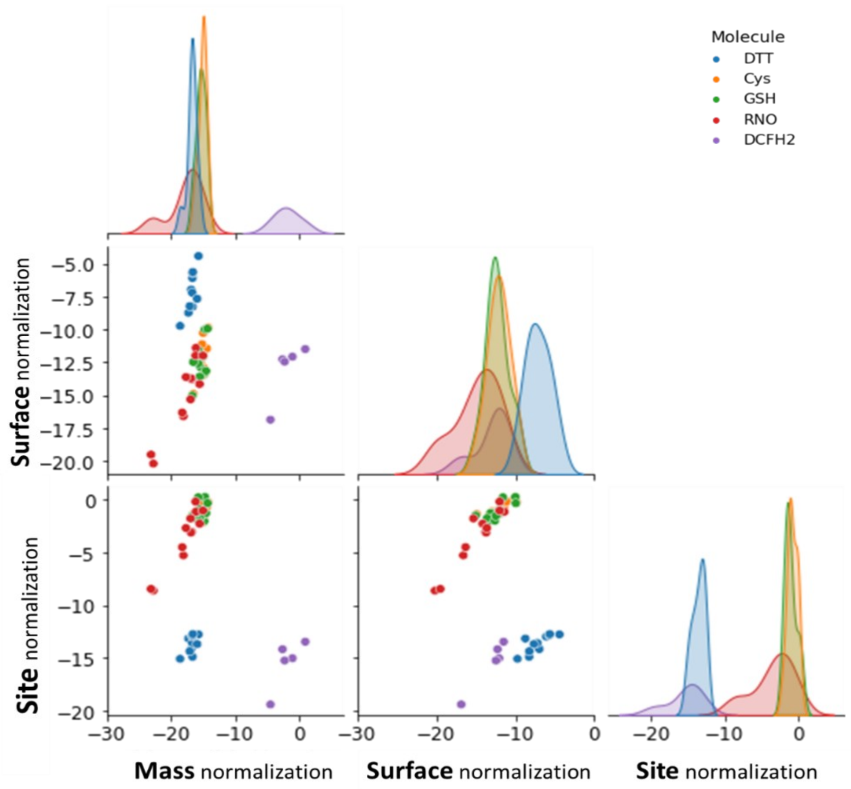

Figure S3. *Pairplot for comparison of normalization methods in the evaluated probe reactions*

In the pairplot, rows and columns represent the x and y axis, respectively. The diagonal subplots provide the probability density function (PDF) for the respective normalization variable and probe molecule. The outcomes of various probe reactions were assessed through the Pearson correlation coefficient, as a linear correlation is anticipated in the reactivity context.

Table S4. *Interference test for DCFH$_2$ assay: results indicating up to 30% interference are denoted by red boxes.*

Dose (μg/mL)	Interference (%)											
	TiO_2 NM-101	TiO_2 NM-105	ZnO NM-110	ZnO NM-111	CeO_2 NM-211	CeO_2 NM-212	SiO_2 NM-200	SiO_2 NM-201	MWCNT NM-400	CB	Fe_2O_3	CuO
0	0	0	0	0	0	0	0	0	0	0	0	0
1.6	0	0	2	2	1	-1	-1	0	6	1	0	3
3.1	2	0	4	4	0	2	-1	2	19	3	4	5
6.3	0	-1	9	12	-1	6	-1	-2	37	6	5	9
12.5	0	1	20	19	0	10	-3	-2	65	30	8	17
25	2	1	20	20	0	17	-2	-1	90	80	16	30
50	-8	0	20	20	0	17	0	-2	99	96	29	42
100	6	9	5	5	8	4	9	8	0	9	11	11

3.5 Raw data of DTT, Cys, GSH and RNO reactions

Table S5. *Raw data for DTT and GSH reactions*

Nanomaterial	DTT conversion (%)		DTT NIOG (0-1)		DTT reaction rate per mass (mol/s·g)		DTT reaction rate per area (umol/s·m2)		DTT OxTOF (s-1)		GSH mol consumed/mg		GSH mol consumed/mol		GSH mol consumed/mol		GSH mol consumed/mol sites	
	x̄	SD	x̄	SD	x̄	SD	x̄	SD	x̄	SD	x̄	SD	x̄	SD	x̄	SD	x̄	SD
**TiO2 NM-101**	37.9	7.0	0.5	0.1	5.8E-8	9.7E-10	2.6E-4	4.3E-6	1.4E-5	1.2E-5	3.6E-7	1.4E-7	2.8E-2	1.1E-2	1.6E-6	6.1E-7	1.3E-1	4.9E-2
**TiO2 NM105**	30.6	9.6	0.4	0.1	4.7E-8	1.3E-8	9.4E-4	2.6E-4	4.3E-5	1.2E-5	2.8E-7	1.9E-7	2.3E-2	1.5E-2	5.7E-6	3.8E-6	2.6E-1	1.7E-1
**ZnO NM110**	0.0	0.0	0.0	0.0	0.0E+0	NA	NA	NA	NA	NA	4.0E-7	4.7E-9	3.2E-2	3.8E-4	4.6E-5	5.4E-7	2.0E+0	2.4E-2
**ZnO NM111**	0.0	0.0	0.0	0.0	0.0E+0	NA	NA	NA	NA	NA	1.4E-7	0.0E+0	1.3E-7	6.1E-9	9.5E-6	0.0E+0	1.2E+0	0.0E+0
**SiO2 NM200**	18.5	12.7	0.2	0.1	3.0E-8	2.1E-8	1.6E-4	1.1E-4	1.2E-4	7.9E-5	5.7E-8	6.2E-8	3.4E-3	3.7E-3	3.0E-7	3.3E-7	2.2E-1	2.4E-1
**SiO2 NM201**	4.6	1.4	0.1	0.0	8.3E-9	2.5E-9	6.0E-5	1.8E-5	1.7E-5	5.3E-6	NA	NA	NA	NA	NA	NA	NA	NA
**CeO2 NM211**	32.2	10.5	0.4	0.1	5.6E-8	1.9E-8	7.4E-4	2.5E-4	7.8E-5	2.6E-5	2.0E-7	1.8E-8	3.4E-2	3.2E-3	2.6E-6	2.4E-7	2.7E-1	2.5E-2
**CeO2 NM212**	42.7	8.7	0.5	0.1	5.7E-8	1.2E-8	2.3E-3	4.6E-4	1.4E-4	2.9E-5	1.6E-7	2.3E-9	2.8E-2	3.9E-4	6.3E-6	9.0E-8	4.0E-1	5.6E-3
**MWCNT NM400**	73.5	6.0	0.9	0.1	1.2E-7	1.1E-8	4.7E-4	4.3E-5	7.0E-5	6.4E-6	4.7E-7	6.8E-9	0.0E+0	0.0E+0	2.0E-6	2.8E-8	2.7E-1	4.0E-3
**MWCNT NM401**	28.1	16.5	0.4	0.2	3.7E-8	3.5E-8	2.7E-4	2.5E-4	3.5E-5	3.3E-5	1.8E-7	7.4E-8	0.0E+0	0.0E+0	1.3E-6	5.3E-7	1.7E-1	7.0E-2
**CuO**	82.6	9.5	1.0	0.0	1.5E-7	3.1E-8	1.2E-2	2.6E-3	3.7E-4	7.8E-5	5.9E-7	2.0E-9	4.7E-2	1.6E-4	4.8E-5	1.6E-7	1.5E+0	5.1E-3
**Carbon black**	86.3	0.2	1.1	0.0	2.0E-7	4.9E-10	6.2E-4	1.6E-6	0.0E+0	0.0E+0	4.1E-7	1.7E-9	0.0E+0	0.0E+0	1.3E-6	5.4E-9	0.0E+0	0.0E+0
**Fe2O3**	30.3	5.5	0.4	0.1	5.7E-8	1.1E-8	3.3E-3	6.3E-4	1.8E-4	3.5E-5	1.4E-7	1.2E-8	2.2E-2	2.0E-3	3.4E-6	3.0E-7	1.3E-1	1.2E-2
**Mn2O3**	33.3	3.0	0.4	0.0	6.1E-8	5.4E-9	3.6E-3	3.2E-4	1.9E-4	1.7E-5	6.5E-8	2.0E-8	1.0E-2	3.2E-3	3.8E-6	1.2E-6	2.1E-1	6.5E-2

Table S6. *Raw data for Cys and RNO reactions*

Nanomaterial	Cys mol consumed/mg		Cys mol consumed/mol		Cys mol consumed/m2		Cys mol consumed/mol sites		RNO mol consumed/mg		RNO mol consumed/mol		RNO mol consumed/m2		RNO mol consumed/mol sites	
	x̄	SD	x̄	SD	x̄	SD	x̄	SD	x̄	SD	x̄	SD	x̄	SD	x̄	SD
TiO2 NM-101	3.2E-7	0.0E+0	2.5E-2	4.2E-18	1.4E-6	0.0E+0	1.1E-1	0.0E+0	1.4E-8	7.1E-9	5.3E-4	2.7E-4	6.2E-8	3.2E-8	5.1E-3	2.6E-3

TiO2 NM105	5.3E-7	2.7E-10	4.3E-2	2.2E-5	1.1E-5	5.5E-9	4.9E-1	2.5E-4	5.1E-8	7.2E-9	1.9E-3	2.7E-4	1.0E-6	1.5E-7	4.7E-2	6.7E-3
ZnO NM110	3.0E-7	4.1E-9	2.5E-2	3.3E-4	3.5E-5	4.7E-7	1.5E+0	2.0E-2	9.8E-8	1.9E-8	7.8E-3	1.5E-3	1.1E-5	2.2E-6	4.9E-1	9.5E-2
ZnO NM111	1.0E-7	5.9E-9	1.7E-2	9.6E-4	7.0E-6	3.9E-7	9.0E-1	5.1E-2	9.4E-8	8.0E-9	7.5E-3	6.4E-4	6.3E-6	5.4E-7	8.1E-1	6.9E-2
SiO2 NM200	6.5E-8	2.6E-8	3.9E-3	1.6E-3	3.4E-7	1.4E-7	2.5E-1	9.9E-2	4.3E-8	4.1E-9	2.6E-3	2.5E-4	2.3E-7	2.2E-8	1.6E-1	1.6E-2
SiO2 NM201	NA	NA	NA	NA	NA	NA	NA	NA	NA	NA	NA	NA	NA	NA	NA	NA
CeO2 NM211	2.3E-7	1.9E-8	4.0E-2	3.2E-3	3.1E-6	2.5E-7	3.2E-1	2.6E-2	1.3E-10	1.4E-18	2.2E-5	2.5E-13	1.7E-9	1.9E-17	1.8E-4	2.0E-12
CeO2 NM212	1.5E-7	0.0E+0	5.1E-2	8.5E-6	5.8E-6	0.0E+0	3.6E-1	0.0E+0	8.6E-11	0.0E+0	1.5E-5	2.1E-21	3.4E-9	0.0E+0	2.1E-4	0.0E+0
MWCNT NM400	4.0E-7	1.0E-9	0.0E+0	0.0E+0	1.7E-6	4.2E-9	2.3E-1	5.8E-4	1.7E-7	2.9E-9	0.0E+0	0.0E+0	7.2E-7	1.2E-8	1.0E-1	1.7E-3
MWCNT NM401	3.4E-7	2.3E-8	0.0E+0	0.0E+0	2.4E-6	1.6E-7	3.2E-1	2.2E-2	1.2E-8	2.0E-8	0.0E+0	0.0E+0	8.3E-8	1.4E-7	1.1E-2	1.9E-2
CuO	6.5E-7	7.1E-9	5.2E-2	5.7E-4	5.3E-5	5.8E-7	1.6E+0	1.8E-2	2.9E-7	0.0E+0	2.2E-2	0.0E+0	2.4E-5	0.0E+0	7.3E-1	0.0E+0
Carbon black	4.6E-7	1.6E-8	0.0E+0	0.0E+0	1.5E-6	5.1E-8	0.0E+0	0.0E+0	1.6E-7	7.9E-10	0.0E+0	0.0E+0	5.0E-7	2.5E-9	0.0E+0	0.0E+0
Fe2O3	2.7E-7	1.4E-10	4.3E-2	2.2E-5	6.6E-6	3.4E-9	2.6E-1	1.3E-4	4.5E-8	2.7E-9	7.3E-3	4.3E-4	1.1E-6	6.6E-8	4.4E-2	2.6E-3
Mn2O3	2.5E-7	3.8E-9	4.0E-2	6.0E-4	1.5E-5	2.2E-7	8.1E-1	1.2E-2	2.1E-8	1.9E-9	1.7E-3	1.5E-4	1.2E-6	1.1E-7	6.7E-2	6.1E-3

4 REFERENCES

1. Rasmussen K, Mast J, Temmerman P jan De, et al. *Titanium Dioxide, NM-100, NM-101, NM-102, NM-103, NM-104, NM-105: Characterisation and Physico- Chemical Properties*.; 2014. doi:10.2788/79554
2. *JRC NANOMATERIALS REPOSITORY. List of Representative Nanomaterials*.; 2016.
3. Kermanizadeh A, Pojana G, Gaiser BK, et al. In vitro assessment of engineered nanomaterials using a hepatocyte cell line: Cytotoxicity, pro-inflammatory cytokines and functional markers. *Nanotoxicology*. 2013;7(3):301-313. doi:10.3109/17435390.2011.653416
4. Singh C, Europäische Kommission Gemeinsame Forschungsstelle Institute for Health and Consumer Protection. *Cerium Dioxide NM-211, NM-212, NM-213, Characterisation and Test Item Preparation JRC Repository: NM-Series of Representative Manufactured Nanomaterials*.; 2014. doi:10.2788/80203
5. Singh C, Friedrichs S, Levin M, et al. *Zinc Oxide NM-110, NM-111, NM-112, NM-113 Characterisation and Test Item Preparation*.; 2011. doi:10.2787/55008
6. Rasmussen K, Mech A, Mast J, et al. *Synthetic Amorphous Silicon Dioxide (NM-200, NM-201, NM-202, NM-203, NM-204): Characterisation and Physico-Chemical Properties JRC*.; 2013. doi:10.2788/57989
7. Guichard Y, Schmit J, Darne C, et al. Cytotoxicity and Genotoxicity of Nanosized and Microsized Titanium Dioxide and Iron Oxide Particles in Syrian Hamster Embryo Cells. *Ann Occup Hyg*. 2012;56(5):631-644. doi:10.1093/annhyg/mes006
8. Life M. *Copper Oxide (Ref. Number: 544868) Specifications*.; 2023. https://www.sigmaaldrich.com/ES/es/product/aldrich/544868
9. Martín-Gómez J, Hidalgo-Carrillo J, Estévez RC, Urbano FJ, Marinas A. Hydrogen photoproduction on TiO2-CuO artificial olive leaves. *Appl Catal A Gen*. 2021;620(March):118178. doi:10.1016/j.apcata.2021.118178
10. Martín-Gómez J, Hidalgo-Carrillo J, Montes V, et al. EPR and CV studies cast further light on the origin of the enhanced hydrogen production through glycerol photoreforming on CuO:TiO2 physical mixtures. *J Environ Chem Eng*. 2021;9(4):105336. doi:10.1016/j.jece.2021.105336

11. Pal AK, Bello D, Budhlall B, Rogers E, Milton DK. Screening for oxidative stress elicited by engineered nanomaterials: Evaluation of acellular DCFH assay. *Dose-Response*. 2012;10(3):308-330. doi:10.2203/dose-response.10-036.Pal

12. *JOINT MEETING OF THE CHEMICALS COMMITTEE AND THE WORKING PARTY ON CHEMICALS, PESTICIDES AND BIOTECHNOLOGY TITANIUM DIOXIDE: SUMMARY OF THE DOSSIER Series on the Safety of Manufactured Nanomaterials No. 73 16th Meeting of the Working Party on Manufactured Nanomaterials*.; 2016.

13. Darne C, Desforges A, Berrada N, et al. A non-damaging purification method: decoupling the toxicity of multi-walled carbon nanotubes and their associated metal impurities. *Environ Sci Nano*. 2019;6(6):1852-1865. doi:10.1039/C8EN01276H

14. Arts JHE, Irfan MA, Keene AM, et al. Case studies putting the decision-making framework for the grouping and testing of nanomaterials (DF4nanoGrouping) into practice. *Regulatory Toxicology and Pharmacology*. 2016;76:234-261. doi:10.1016/j.yrtph.2015.11.020

15. Rasmussen K, Mast J, Temmerman PJ, Verleysen E, Waegeneers N, Steen F. *Multi-Walled Carbon Nanotubes, NM-400, NM-401, NM-402, NM-403: Characterisation and Physico-Chemical Properties In: NM-Series of Representative Manufactured Nanomaterials*.; 2014. doi:10.2788/10753

16. Pal AK, Bello D, Budhlall B, Rogers E, Milton DK. Screening for oxidative stress elicited by engineered nanomaterials: Evaluation of acellular DCFH assay. *Dose-Response*. 2012;10(3):308-330. doi:10.2203/dose-response.10-036.Pal

17. Foucaud L, Wilson MR, Brown DM, Stone V. Measurement of reactive species production by nanoparticles prepared in biologically relevant media. *Toxicol Lett*. 2007;174(1-3):1-9. doi:10.1016/j.toxlet.2007.08.001

18. Sauvain JJ, Rossi MJ, Riediker M. Comparison of three acellular tests for assessing the oxidation potential of nanomaterials. *Aerosol Science and Technology*. 2013;47(2):218-227. doi:10.1080/02786826.2012.742951

19. Gliga AR, Skoglund S, Odnevall Wallinder I, Fadeel B, Karlsson HL. Size-dependent cytotoxicity of silver nanoparticles in human lung cells: The role of cellular uptake, agglomeration and Ag release. *Part Fibre Toxicol*. 2014;11(1):1-17. doi:10.1186/1743-8977-11-11

20. Ag Seleci D, Tsiliki G, Werle K, et al. Determining nanoform similarity via assessment of surface reactivity by abiotic and in vitro assays. *NanoImpact*. 2022;26(September 2021):100390. doi:10.1016/j.impact.2022.100390
21. Alcolea-Rodriguez V, Dumit VI, Ledwith R, Portela R, Bañares MA, Haase A. Differentially Induced Autophagy by Engineered Nanomaterial Treatment Has an Impact on Cellular Homeostasis and Cytotoxicity. doi:10.1021/acs.nanolett.4c01573

5 ACKNOWLEDGMENTS

The authors are grateful for the financial support from the European Commission H2020 project NanoInformaTIX (grant agreement number 814426), and to the German Federal Institute for Risk Assessment for Victor Alcolea-Rodriguez's stay, which was partially funded by European Commission H2020 project HARMLESS (grant agreement number 953183). We are also indebted to F.J. R. Vasques for his support with experimental work at ICP-CSIC.

All authors have given approval to the final version of the manuscript. The authors declare no competing financial interest.
